# Continuing Professional Development — Medical Imaging

**DOI:** 10.1002/jmrs.70058

**Published:** 2026-02-27

Maximise your continuing professional development (CPD) by reading the selected article and answering the five questions. Please remember to self‐claim your CPD and retain your supporting evidence. Answers will be available via the QR code and published in JMRS—Volume 73, Issue 4, December 2026.

## Impact of Ceiling Suspended Shield Size on Primary Operator Radiation Dose During Coronary Angiography and Intervention

James A. Crowhurst, Elizabeth Andersen, Michael Savage, Jason Tse, Dale Murdoch, Darren Walters, Rustem Dautov, https://doi.org/10.1002/jmrs.70042.

1. In this study, which two metrics were used to quantify the procedural radiation dose?
  - Fluoroscopy time and air kerma
  - Fluoroscopy time and effective dose
  - Fluoroscopy time and kerma area product
  - Air kerma and kerma area product
2. What was the primary difference in operator shielding between the two angiography rooms described in the study?
  - Presence of lower‐body table‐attached shielding
  - Size of the ceiling‐suspended lead‐acrylic shield
  - Thickness of the operator lead apron
  - Placement and orientation of movable lead shields
3. Applying angiographic radiation physics principles to the procedural context described in the study, which source most accurately explains staff radiation exposure during procedures?
  - Direct irradiation from the primary X‐ray beam
  - Leakage through defects in personal protective equipment
  - Inadequate positioning of ceiling‐suspended shielding
  - Scatter radiation originating from the patient
4. When comparing the two angiography rooms, which radiation‐related outcome showed a statistically significant difference indicating a measurable benefit of the shielding configuration?
  - Fluoroscopy time
  - Kerma area product
  - Operator dose
  - Both kerma area product and operator dose
5. Based on the study's findings and established radiation protection principles, which approach would be most effective in minimising staff radiation exposure during angiographic procedures?
  - Maximising distance from the patient while optimising the placement and use of protective shielding
  - Increasing room size to allow greater separation between staff and the X‐ray source
  - Limiting total time spent in the angiography room regardless of staff position
  - Selecting imaging protocols solely based on patient body habitus

## Answers

**Figure:**
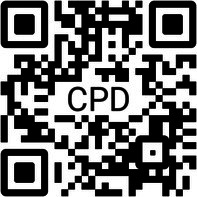
Scan this QR code to find the answers.
